# Supplementary material for: Genome-Wide Investigation of the NF-X1 Gene Family in Populus trichocarpa Expression Profiles during Development and Stress
Source: Int J Mol Sci. 2021 Apr 28;22(9):4664. doi: 10.3390/ijms22094664 (PMC8124260; doi:10.3390/ijms22094664)
Supplement: Supplementary file 1 [file ijms-22-04664-s001.zip › ijms-1201645-supplementary.pdf]

Table S1. Primer sequences used RT-PCR

Gene Name	Forward primers	Reverse primers
PtrNF-X1-1	TTGTTGCTATGCCATCCCGT	GGCAACCTCTAACACTCCCC
PtrNF-X1-2	ATGGCTGCTCGTTCATGTGG	ACCTTCTCCTCCAGGTCTTTGG
PtrNF-X1-3	GTAGCAGGTCCAGATTCCCG	CTCGAAGGTGGGTTTGGGTT
PtrNF-X1-4	GCGCACCTTGTTTGGTTTCT	GGAAGTTCTCCTTAACTTCTTCCCA

Table S2. Accession numbers of gene

Order	Species	abb.	Nr	Accession numbers	Name
1	*Amaranthus hypochondriacus*	*Ah*	3	*AHYPO_010689-RA*	*AhNF-X1-1*
				AHYPO_013985-RA	*AhNF-X1-2*
				AHYPO_014930-RA	*AhNF-X1-3*
2	*Amborella trichopoda*	*Atr*	1	*evm_27.model.AmTr_v1.0_scaffold 00060.63*	*AtrNF-X1-1*
3	*Ananas comosus*	*Ac*	2	*Aco002964.1*	*AcNF-X1-1*
				Aco021291.1	*AcNF-X1-2*
4	*Aquilegia coerulea*	*Aco*	3	*Aqcoe1G302400.1*	*AcoNF-X1-1*
				Aqcoe1G420000.1	*AcoNF-X1-2*
				Aqcoe3G241800.1	*AcoNF-X1-3*
5	*Arabidopsis thaliana*	*At*	2	*AT1G10170.1*	*AtNF-X1-1*
				AT5G05660.1	*AtNF-X1-2*
6	*Boechera stricta*	*Bs*	3	*Bostr.13129s0405.1*	*BsNF-X1-1*
				Bostr.13671s0471.1	*BsNF-X1-2*
				Bostr.15697s0430.1	*BsNF-X1-3*
7	*Brachypodium distachyon*	*Bd*	2	*Bradi1g44270.2*	*BdNF-X1-1*
				Bradi2g00226.1	*BdNF-X1-2*
8	*Brachypodium stacei*	*Bst*	2	*Brast01G414700.1*	*BstNF-X1-1*
				Brast07G091900.1	*BstNF-X1-2*
9	*Capsella rubella*	*Cr*	2	*Carubv10003051m*	*CrNF-X1-1*
				Carubv10008145m	*CrNF-X1-2*
10	*Carica papaya*	*Cp*	1	*evm.model.supercontig_19.173*	*CpNF-X1-1*
11	*Chlamydomonas reinhardtii*	*Cre*	1	*Cre03.g163200.t1.1*	*CreNF-X1-1*

12	*Citrus clementina*	*Cc*	2	*Ciclev10018607m*	*CcNF-X1-1*
				Ciclev10030684m	*CcNF-X1-2*
13	*Citrus sinensis*	*Cs*	6	*orange1.1g001376m*	*CsNF-X1-1*
				orange1.1g007271m	*CsNF-X1-2*
				orange1.1g009037m	*CsNF-X1-3*
				orange1.1g011784m	*CsNF-X1-4*
				orange1.1g011809m	*CsNF-X1-5*
				orange1.1g011815m	*CsNF-X1-6*
14	*Coccomyxa subellipsoidea C-169*	*Cos*	1	*65191*	*CosNF-X1-1*
15	*Daucus carota*	*Dc*	2	*DCAR_003534*	*DcNF-X1-1*
				DCAR_024827	*DcNF-X1-2*
16	*Eucalyptus grandis*	*Eg*	3	*Eucgr.H00552.1*	*EgNF-X1-1*
				Eucgr.J00206.1	*EgNF-X1-2*
				Eucgr.K02284.1	*EgNF-X1-3*
17	*Eutrema salsugineum*	*Es*	3	*Thhalv10006632m*	*EsNF-X1-1*
				Thhalv10006708m	*EsNF-X1-2*
				Thhalv10012642m	*EsNF-X1-3*
18	*Fragaria vesca*	*Fv*	2	*mrna09346.1-v1.0-hybrid*	*FvNF-X1-1*
				mrna20971.1-v1.0-hybrid	*FvNF-X1-2*
19	*Glycine max*	*Gm*	9	*Glyma.07G104700.1*	*GmNF-X1-1*
				Glyma.07G104700.2	*GmNF-X1-2*
				Glyma.08G055900.1	*GmNF-X1-3*
				Glyma.09G173000.1	*GmNF-X1-4*
				Glyma.11G230200.1	*GmNF-X1-5*
				Glyma.18G027000.1	*GmNF-X1-6*
				Glyma.18G027000.2	*GmNF-X1-7*
				Glyma.18G027000.3	*GmNF-X1-8*
				Glyma.18G027000.4	*GmNF-X1-9*
20	*Gossypium raimondii*	*Gr*	3	*Gorai.006G058600.1*	*GrNF-X1-1*
				Gorai.010G197800.1	*GrNF-X1-2*
				Gorai.010G197800.2	*GrNF-X1-3*
21	*Linum usitatissimum*	*Lu*	4	*Lus10025071*	*LuNF-X1-1*
				Lus10028286	*LuNF-X1-2*
				Lus10034470	*LuNF-X1-3*
				Lus10040200	*LuNF-X1-4*
22	*Malus domestica*	*Md*	3	*MDP0000239523*	*MdNF-X1-1*
				MDP0000280911	*MdNF-X1-2*
				MDP0000603846	*MdNF-X1-3*
23	*Manihot esculenta*	*Me*	3	*Manes.06G003200.1*	*MeNF-X1-1*
				Manes.06G003200.2	*MeNF-X1-2*
				Manes.10G012200.1	*MeNF-X1-3*

24	*Marchantia polymorpha*	*Mp*	4	*Mapoly0036s0109.1*	*MpNF-X1-1*
				Mapoly0036s0109.2	*MpNF-X1-2*
				Mapoly0036s0109.3	*MpNF-X1-3*
				Mapoly0154s0047.1	*MpNF-X1-4*
25	*Medicago truncatula*	*Mt*	4	*Medtr3g070780.1*	*MtNF-X1-1*
				Medtr3g070780.2	*MtNF-X1-2*
				Medtr4g027040.1	*MtNF-X1-3*
				Medtr6g071140.1	*MtNF-X1-4*
26	*Mimulus guttatus*	*Mg*	2	*Migut.D01221.1*	*MgNF-X1-1*
				Migut.F00683.1	*MgNF-X1-2*
27	*Musa acuminata*	*Ma*	2	*GSMUA_Achr2T18970_001*	*MaNF-X1-1*
				GSMUA_Achr7T12440_001	*MaNF-X1-2*
28	*Oropetium thomaeum*	*Ot*	1	*Oropetium_20150105_07174A*	*OtNF-X1-1*
29	*Oryza sativa subsp. japonica*	*Os*	2	*LOC_Os01g06550.1*	*OsNF-X1-1*
				LOC_Os06g14190.1	*OsNF-X1-2*
30	*Panicum hallii*	*Ph*	2	*Pahal.E04060.1*	*PhNF-X1-1*
				Pahal.E04060.2	*PhNF-X1-2*
31	*Panicum virgatum*	*Pv*	3	*Pavir.4KG191400.1*	*PvNF-X1-1*
				Pavir.5KG080800.1	*PvNF-X1-2*
				Pavir.5NG084900.1	*PvNF-X1-3*
32	*Phaseolus vulgaris*	*Pvu*	2	*Phvul.001G243400.1*	*PvuNF-X1-1*
				Phvul.002G331600.1	*PvuNF-X1-2*
33	*Physcomitrella patens*	*Pp*	1	*Pp3c2_24250V3.1*	*PpNF-X1-1*
34	*Populus euphratica*	*Pe*	3	*CCG002829.1*	*PeNF-X1-1*
				CCG014737.1	*PeNF-X1-2*
				CCG020445.2	*PeNF-X1-3*
35	*Populus trichocarpa*	*Ptr*	4	*Potri.008G068500.1*	*PtrNF-X1-1*
				Potri.010G188700.1	*PtrNF-X1-2*
				Potri.012G043700.1	*PtrNF-X1-3*
				Potri.015G034500.1	*PtrNF-X1-4*
36	*Prunus persica*	*Ppe*	2	*Prupe.2G250200.1*	*PpeNF-X1-1*
				Prupe.5G236400.1	*PpeNF-X1-2*
37	*Salix purpurea*	*Sp*	5	*SapurV1A.0061s0270.1*	*SpNF-X1-1*
				SapurV1A.0061s0270.3	*SpNF-X1-2*
				SapurV1A.0082s0290.1	*SpNF-X1-3*
				SapurV1A.0665s0040.1	*SpNF-X1-4*
				SapurV1A.0665s0040.2	*SpNF-X1-5*
38	*Setaria italica*	*Si*	2	*Seita.4G110900.1*	*SiNF-X1-1*
				Seita.5G112600.1	*SiNF-X1-2*
39	*Setaria viridis*	*Sv*	3	*Sevir.4G111000.1*	*SvNF-X1-1*
				Sevir.5G109300.1	*SvNF-X1-2*

				Sevir.5G109300.2	SvNF-X1-3
40	Sisymbrium irio	Sir	2	676723276	SirNF-X1-1
				676738184	SirNF-X1-2
41	Solanum lycopersicum	Sl	2	Solyc03g118420.2.1	SlNF-X1-1
				Solyc06g051570.2.1	SlNF-X1-2
42	Solanum tuberosum	St	2	PGSC0003DMT400036773	StNF-X1-1
				PGSC0003DMT400036774	StNF-X1-2
43	Sorghum bicolor	Sb	3	Sobic.003G062300.1	SbNF-X1-1
				Sobic.004G354301.1	SbNF-X1-2
				Sobic.010G104100.1	SbNF-X1-3
44	Sphagnum fallax	Sf	2	Sphfalx0130s0063.1	SfNF-X1-1
				Sphfalx0218s0019.1	SfNF-X1-2
45	Spirodela polyrhiza	Spo	2	Spipo23G0035500	SpoNF-X1-1
				Spipo2G0021300	SpoNF-X1-2
46	Theobroma cacao	Tc	8	Thecc1EG012484t1	TcNF-X1-1
				Thecc1EG017029t1	TcNF-X1-2
				Thecc1EG017029t2	TcNF-X1-3
				Thecc1EG017029t3	TcNF-X1-4
				Thecc1EG017029t4	TcNF-X1-5
				Thecc1EG042496t1	TcNF-X1-6
				Thecc1EG042496t2	TcNF-X1-7
				Thecc1EG042496t3	TcNF-X1-8
47	Trifolium pratense	Tp	2	Tp57577_TGAC_v2_mRNA11567	TpNF-X1-1
				Tp57577_TGAC_v2_mRNA23077	TpNF-X1-2
48	Triticum aestivum	Ta	1	Traes_3AS_496047B73.2	TaNF-X1-1
49	Vitis vinifera	Vv	2	GSVIVT01008482001	VvNF-X1-1
				GSVIVT01014100001	VvNF-X1-2
50	Volvox carteri	Vc	1	Vocar.0005s0190.1	VcNF-X1-1
51	Zea mays	Zm	4	GRMZM2G087787_T01	ZmNF-X1-1
				GRMZM2G139369_T01	ZmNF-X1-2
				GRMZM2G307823_T01	ZmNF-X1-3
				GRMZM2G409627_T01	ZmNF-X1-4
52	Zostera marina	Zma	2	Zosma28g00280.1	ZmaNF-X1-1
				Zosma32g00890.1	ZmaNF-X1-2

Table S3. Amino acid conserved domain

Gene ID	Accession	From	To	Short name
Q#1 - >AtNF-X1-1	cd16696	222	279	RING-CH-C4HC3_NFX1
Q#1 - >AtNF-X1-1	cd06008	503	550	NF-X1-zinc-finger
Q#1 - >AtNF-X1-1	cd06008	380	428	NF-X1-zinc-finger
Q#1 - >AtNF-X1-1	cd06008	601	653	NF-X1-zinc-finger
Q#1 - >AtNF-X1-1	cd06008	448	489	NF-X1-zinc-finger
Q#1 - >AtNF-X1-1	cd06008	658	705	NF-X1-zinc-finger
Q#1 - >AtNF-X1-1	cd06008	312	373	NF-X1-zinc-finger
Q#1 - >AtNF-X1-1	cd06008	562	622	NF-X1-zinc-finger
Q#1 - >AtNF-X1-1	cl00297	888	963	R3H
Q#1 - >AtNF-X1-1	cd06008	749	798	NF-X1-zinc-finger
Q#1 - >AtNF-X1-1	pfam12273	58	134	RCR
Q#1 - >AtNF-X1-1	cl35953	86	215	rne
Q#1 - >AtNF-X1-1	cl10082	711	766	NF-X1-zinc-finger
Q#2 - >AtNF-X1-2	cd16697	82	149	RING-CH-C4HC3_NFXL1
Q#2 - >AtNF-X1-2	cd06008	424	472	NF-X1-zinc-finger
Q#2 - >AtNF-X1-2	cd06008	344	393	NF-X1-zinc-finger
Q#2 - >AtNF-X1-2	cd06008	237	285	NF-X1-zinc-finger
Q#2 - >AtNF-X1-2	cd06008	290	330	NF-X1-zinc-finger
Q#2 - >AtNF-X1-2	cd06008	397	445	NF-X1-zinc-finger
Q#2 - >AtNF-X1-2	cd06008	183	231	NF-X1-zinc-finger
Q#2 - >AtNF-X1-2	cl10082	631	672	NF-X1-zinc-finger
Q#3 - >OsNF-X1-1	cl17238	109	160	RING_Ubox
Q#3 - >OsNF-X1-1	cd06008	439	487	NF-X1-zinc-finger
Q#3 - >OsNF-X1-1	cd06008	412	460	NF-X1-zinc-finger
Q#3 - >OsNF-X1-1	cd06008	359	408	NF-X1-zinc-finger
Q#3 - >OsNF-X1-1	cd06008	252	300	NF-X1-zinc-finger
Q#3 - >OsNF-X1-1	cd06008	305	345	NF-X1-zinc-finger
Q#3 - >OsNF-X1-1	cl10082	659	700	NF-X1-zinc-finger
Q#3 - >OsNF-X1-1	cl10082	723	774	NF-X1-zinc-finger
Q#4 - >OsNF-X1-2	cd16696	1	54	RING-CH-C4HC3_NFX1
Q#4 - >OsNF-X1-2	cd06008	279	327	NF-X1-zinc-finger
Q#4 - >OsNF-X1-2	cd06008	157	205	NF-X1-zinc-finger
Q#4 - >OsNF-X1-2	cd06008	374	426	NF-X1-zinc-finger
Q#4 - >OsNF-X1-2	cd06008	221	264	NF-X1-zinc-finger
Q#4 - >OsNF-X1-2	cl10082	431	479	NF-X1-zinc-finger
Q#4 - >OsNF-X1-2	cl10082	87	150	NF-X1-zinc-finger
Q#4 - >OsNF-X1-2	cl10082	484	510	NF-X1-zinc-finger
Q#5 - >PtrNF-X1-1	cd06008	228	272	NF-X1-zinc-finger

Q#6 - >PtrNF-X1-2	cl17238	90	157	RING_Ubox
Q#6 - >PtrNF-X1-2	cd06008	431	479	NF-X1-zinc-finger
Q#6 - >PtrNF-X1-2	cd06008	351	400	NF-X1-zinc-finger
Q#6 - >PtrNF-X1-2	cd06008	404	452	NF-X1-zinc-finger
Q#6 - >PtrNF-X1-2	cd06008	244	284	NF-X1-zinc-finger
Q#6 - >PtrNF-X1-2	cd06008	299	340	NF-X1-zinc-finger
Q#6 - >PtrNF-X1-2	cd06008	190	238	NF-X1-zinc-finger
Q#6 - >PtrNF-X1-2	cd06008	641	682	NF-X1-zinc-finger
Q#6 - >PtrNF-X1-2	cl10082	705	756	NF-X1-zinc-finger
Q#7 - >PtrNF-X1-3	cd16696	122	179	RING-CH-C4HC3_NFX1
Q#7 - >PtrNF-X1-3	cd06008	402	450	NF-X1-zinc-finger
Q#7 - >PtrNF-X1-3	cd06008	280	328	NF-X1-zinc-finger
Q#7 - >PtrNF-X1-3	cd06008	344	387	NF-X1-zinc-finger
Q#7 - >PtrNF-X1-3	cd06008	498	541	NF-X1-zinc-finger
Q#7 - >PtrNF-X1-3	cl00297	781	854	R3H
Q#7 - >PtrNF-X1-3	cd06008	646	695	NF-X1-zinc-finger
Q#7 - >PtrNF-X1-3	cd06008	555	603	NF-X1-zinc-finger
Q#7 - >PtrNF-X1-3	cd06008	212	273	NF-X1-zinc-finger
Q#7 - >PtrNF-X1-3	cl10082	460	519	NF-X1-zinc-finger
Q#7 - >PtrNF-X1-3	cl10082	608	663	NF-X1-zinc-finger
Q#8 - >PtrNF-X1-4	cd16696	122	179	RING-CH-C4HC3_NFX1
Q#8 - >PtrNF-X1-4	cd06008	402	450	NF-X1-zinc-finger
Q#8 - >PtrNF-X1-4	cd06008	280	328	NF-X1-zinc-finger
Q#8 - >PtrNF-X1-4	cd06008	344	387	NF-X1-zinc-finger
Q#8 - >PtrNF-X1-4	cl00297	750	823	R3H
Q#8 - >PtrNF-X1-4	cd06008	212	273	NF-X1-zinc-finger
Q#8 - >PtrNF-X1-4	cd06008	615	664	NF-X1-zinc-finger
Q#8 - >PtrNF-X1-4	cd06008	524	572	NF-X1-zinc-finger
Q#8 - >PtrNF-X1-4	cl17169	858	915	RRM_SF

Table S4. Amino acid sequence encode and SeqLogo of the Motif

Motif ID	Amino acid sequence encode	SeqLogo
Motif_1	FDALVDMDPRLVVSFLDLPREADISSLVLRFGGECELVWLNDKNALAVFN	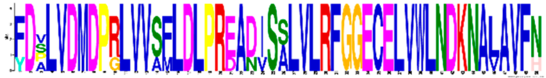
Motif_2	VLADAFDITPPNLEALHFGENSAVTELIGDLYRRDPKWVLAVEERCKYLV	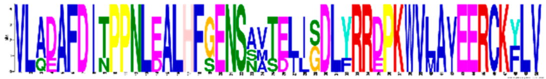
Motif_3	MICYDMVRRSAPVWSCSSCFSIFHLNCIK	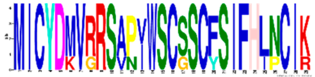
Motif_4	RGTTSGLKIHVFCPMLKDKRDAVRLIAERWKVAIYSAGWEPKRFIVIHAT	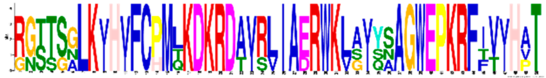
Motif_5	CHFGDCPPCSVPVAKECVGGHVILGNIPCGSRDIRCNKLCGKTRQCGLHA	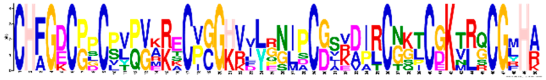
Motif_6	CPDVRCEFLVTISCSCGRMTASVPCDAGG	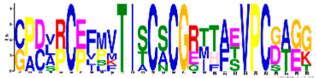
Motif_7	WARAPTSVDLIAEKNQGFNWRCPGCQSVQLTSLKDIRYVCF	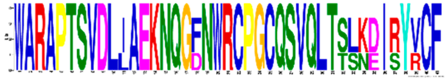
Motif_8	FSCNNICKKSLDCGIHSCKQICHDGPCPPCNARGVYKCSCG	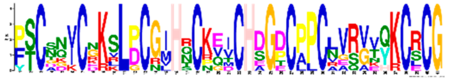
Motif_9	QVLVNASCFCKKKTEVVLCGDMAVKGEVKAEDGVFSCNSTCGKMLGCGNH	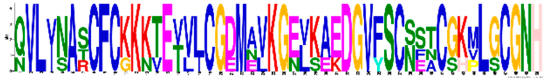
Motif_10	GGYNDTILEASILHKLPAPLQPVESSGKKIPLGQRKFMCDDECAKFERKR	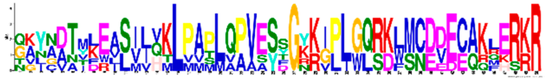

Table S5. **Collinear genes among same species**

Ptr-Ptr
PtrNF-X1-1——PtrNF-X1-2
PtrNF-X1-3——PtrNF-X1-4

Table S6. **Collinear genes among different species**

Ptr-At	Ptr-Os
PtrNF-X1-1，PtrNF-X1-2-AtNF-X1-2	*PtrNF-X1-1-OsNF-X1-1*

Table S7. **Information of functional elements in *NF-X1* genes**

GeneID	Element	Sequence	Description
	MBS	CAACTG	Drought inducibility
	MBS	CAACTG	Drought inducibility
	P-box	CCTTTTG	Gibberellin responsiveness
	CAT-box	GCCACT	Meristem expression
	CAT-box	GCCACT	Meristem expression
	TGACG-motif	TGACG	MeJA responsiveness
	CGTCA-motif	CGTCA	MeJA responsiveness
PtrNF-X1-1	ABRE	GACACGTACGT	Abscisic acid responsiveness
	ABRE	TACGTGTC	Abscisic acid responsiveness
	ABRE	ACGTG	Abscisic acid responsiveness
	ARE	AAACCA	Anaerobic induction
	ARE	AAACCA	Anaerobic induction
	TCA-element	TCAGAAGAGG	Salicylic acid responsiveness
	TCA-element	CCATCTTTTT	Salicylic acid responsiveness
	GCN4_motif	TGAGTCA	Endosperm expression
	LTR	CCGAAA	Low temperature responsiveness
	TCA-element	TCAGAAGAGG	Salicylic acid responsiveness
	TCA-element	CCATCTTTTT	Salicylic acid responsiveness
	ARE	AAACCA	Anaerobic induction
PtrNF-X1-2	ARE	AAACCA	Anaerobic induction
	ABRE	ACGTG	Abscisic acid responsiveness
	ABRE	CACGTG	Abscisic acid responsiveness
	ABRE	ACGTG	Abscisic acid responsiveness
	RY-element	CATGCATG	Seed specific regulation
	GC-motif	CCCCCG	Anoxic specific inducibility

	TGACG-motif	TGACG	MeJA responsiveness
	CAT-box	GCCACT	Meristem expression
	TC-rich repeats	GTTTTCTTAC	Defense and stress responsiveness
PtrNF-X1-3	ARE	AAACCA	Anaerobic induction
	ABRE	CGCACGTGTC	Abscisic acid responsiveness
	ABRE	CACGTG	Abscisic acid responsiveness
	ABRE	ACGTG	Abscisic acid responsiveness
	CGTCA-motif	CGTCA	MeJA responsiveness
	TGA-element	AACGAC	Auxin responsive
	TC-rich repeats	GTTTTCTTAC	Defense and stress responsiveness
	TC-rich repeats	GTTTTCTTAC	Defense and stress responsiveness
	TC-rich repeats	ATTCTCTAAC	Defense and stress responsiveness
	TC-rich repeats	ATTCTCTAAC	Defense and stress responsiveness
PtrNF-X1-4	LTR	CCGAAA	Low temperature responsiveness
	TGA-element	AACGAC	Auxin responsive
	RY-element	CATGCATG	Seed specific regulation
	ARE	AAACCA	Anaerobic induction
	ARE	AAACCA	Anaerobic induction
	ARE	AAACCA	Anaerobic induction
	TCA-element	CCATCTTTTT	Salicylic acid responsiveness
	ABRE	CACGTG	Abscisic acid responsiveness
	ABRE	ACGTG	Abscisic acid responsiveness

Table S8. The number of functional elements in *NF-X1* genes

Gene ID	MeJA-responsiveness	salicylic acid responsiveness	abscisic acid responsiveness	drought-inducibility	defense and stress responsiveness	low-temperature responsiveness	anoxic specific inducibility	anaerobic induction	Meristem expression	gibberellin responsiveness	auxin-responsive	seed-specific regulation	endosperm expression
PtrNF-X1-1	2	2	3	2	/	/	/	2	2	1	/	/	1
PtrNF-X1-2	/	2	3	/	/	1	1	2	/	/	/	1	/
PtrNF-X1-3	2	/	3	/	1	/	/	1	1	/	1	/	/
PtrNF-X1-4	/	1	2	/	4	1	/	3	/	/	1	1	/

Table S9. **Expression of *NF-X1* genes in different plant tissues**

Gene ID	Twigs-Non-Girdled	Flowers-Dormant	Flowers-Expanded	Leaves-Mature	Leaves-Mature 2	Flowers-Expanding	Suckers-Whole-Sucker	Petiole-Mature	Buds-Prechilling	Buds-Dormant	Leaves-Freshly-Expanded	Leaves-Non-Girdled	Leaves-Girdled	Seeds-Mature	Leaves-Young-Expanding	Cambium-Phloem-Dormant
PtrNF-X1-1	0.734	-0.072	-0.890	-0.135	-0.438	0.466	0.465	0.414	0.812	0.496	-0.322	-1.783	-0.368	-0.448	0.322	0.104
PtrNF-X1-2	-0.079	0.230	0.141	-0.199	-0.062	-0.078	-0.102	-0.054	0.111	0.425	0.027	0.549	-0.126	0.014	-0.078	0.163
PtrNF-X1-3	-0.355	-0.638	0.079	0.669	0.645	-0.268	-0.070	0.185	-0.450	-0.511	-0.190	1.037	0.507	-0.215	-0.529	-0.583
PtrNF-X1-4	0.111	0.102	0.238	-0.082	-0.106	0.019	-0.182	-0.345	0.108	0.310	-0.181	-0.014	0.377	0.249	0.011	1.294

Table S10. Expression of *NF-X1* genes in different treatments

GeneID	Leaves-Beetle-Damaged	Leaves-Mechanical-Damage	Leaves-Drought	Roots-Drought
PtrNF-X1-1	-0.720	-0.364	-0.606	1.590
PtrNF-X1-2	0.451	0.288	1.285	0.403
PtrNF-X1-3	0.489	-0.003	-0.319	-0.601
PtrNF-X1-4	-0.165	0.055	-0.244	0.323
